# Radial Head Arthroplasty Versus Open Reduction and Internal Fixation for Mason Type III and IV Fractures: A Systematic Review and Meta-Analysis

**DOI:** 10.7759/cureus.95135

**Published:** 2025-10-22

**Authors:** Abdelfatah M Elsenosy, Eslam Hassan, Mustafa Al-Alawi, Ahmed S Yousef, Radwa A Delewar

**Affiliations:** 1 Trauma and Orthopaedics, University Hospital Dorset, Poole, GBR; 2 Trauma and Orthopaedics, Poole General Hospital, Poole, GBR; 3 Pharmacy, Alexandria University, Alexandria, EGY

**Keywords:** elbow trauma, mason classification, meta-analysis, open reduction internal fixation, radial head arthroplasty, radial head fracture, systematic review

## Abstract

The optimal management of Mason type III and IV radial head fractures remains controversial. This systematic review and meta-analysis compared outcomes of radial head arthroplasty (RHA) and open reduction and internal fixation (ORIF). A comprehensive search identified eight cohort studies involving 457 patients. Reported outcomes included range of motion (ROM), Mayo Elbow Performance Score (MEPS), Disabilities of the Arm, Shoulder, and Hand (DASH) score, and complication rates, with data synthesized using pooled effect estimates and heterogeneity assessed through the I² statistic. RHA was associated with significantly improved elbow extension, higher MEPS scores, and lower complication rates compared with ORIF, particularly in cases of severe comminution or Mason type IV fractures, whereas ORIF demonstrated comparable long-term ROM and DASH scores, especially in younger patients with less complex fracture patterns. Overall, RHA appears to provide superior functional outcomes and fewer complications in appropriately selected patients, while ORIF remains a reasonable option for younger individuals with simpler fracture configurations. Treatment decisions should be tailored to fracture severity, patient factors, and surgeon expertise, and further randomized controlled trials are required to establish definitive guidelines for managing complex radial head fractures.

## Introduction and background

Radial head fractures are common injuries in adults, accounting for nearly one-third of all elbow fractures, most often resulting from falls onto an outstretched hand or direct trauma [1]. The Mason classification remains the most widely used system for categorizing these fractures: type I (non-displaced), type II (displaced with minimal comminution), type III (comminuted involving the entire radial head), and type IV (radial head fracture associated with elbow dislocation) [2]. While Mason type I and II fractures are usually managed conservatively or with internal fixation, optimal treatment of type III and IV fractures remains controversial due to their complexity and frequent association with ligamentous injuries [3,4]. ORIF can preserve native anatomy but becomes technically challenging in fractures with significant comminution, often leading to loss of reduction, nonunion, stiffness, or post-traumatic arthritis [5-7], and type IV fractures carry additional risks due to associated instability and concomitant coronoid or ligamentous injuries [6]. Radial head arthroplasty (RHA) has gained popularity as an alternative in cases of irreparable comminution, offering restoration of elbow kinematics and stability, though it is not without complications such as heterotopic ossification, overstuffing, and implant loosening [8,9]. Comparative studies suggest that RHA may provide better short- to mid-term outcomes than ORIF, with improved Mayo Elbow Performance Scores (MEPS), greater range of motion (ROM), and lower complication rates [10,11], yet ORIF remains a valid option in younger patients with less comminuted fracture patterns where anatomical reconstruction is feasible [12]. The choice between RHA and ORIF depends on fracture complexity and patient-specific factors, with some studies reporting equivalent long-term outcomes [13] and others favoring RHA for earlier recovery and reduced revision rates [14]. These conflicting results likely reflect heterogeneity in patient populations, fracture patterns, surgical techniques, and outcome measures [15]. Given these uncertainties, this systematic review and meta-analysis was conducted to compare clinical and functional outcomes between RHA and ORIF for Mason type III and IV radial head fractures.

## Review

Methods

Search Strategy

A comprehensive literature search was conducted in PubMed, Embase, Scopus, and the Cochrane Library to identify studies comparing RHA with open reduction and internal fixation (ORIF) for the treatment of Mason type III and IV radial head fractures. The search was limited to articles published within the last 10 years (January 2015 to May 2025). A combination of Medical Subject Headings (MeSH) and free-text terms was used, including “radial head arthroplasty”, “open reduction and internal fixation”, “Mason fracture”, “radial head fracture”, and “elbow fracture”. Reference lists of included studies and relevant reviews were manually screened to identify additional eligible articles. This review was conducted and reported in accordance with the Preferred Reporting Items for Systematic Reviews and Meta-Analyses (PRISMA) guidelines.

Inclusion and Exclusion Criteria

The inclusion criteria comprised comparative studies evaluating RHA versus ORIF, studies with adult patients (≥18 years) with Mason type III or IV radial head fractures, studies that reported at least one clinical outcome (MEPS, DASH, ROM, or complication rate), and studies published in English in peer-reviewed journals. The study excluded case reports, review articles, biomechanical or cadaveric studies; studies without a comparative group or involving fracture types other than Mason type III/IV; studies with insufficient data or incomplete outcome reporting; and duplicate publications from the same patient cohort.

Outcome Measures

The primary outcomes were elbow function, assessed using the MEPS; disability level, measured using the Disabilities of the Arm, Shoulder, and Hand (DASH) score; ROM (flexion, extension, supination, pronation); and overall complication rates. Secondary outcomes included the incidence of revision surgery and prosthesis-related complications, when reported.

Data Extraction and Quality Assessment

Data were independently extracted by the reviewers using a standardized form to collect study characteristics, patient demographics, interventions, outcome measures, and follow-up durations. The methodological quality of included studies was evaluated using the Newcastle-Ottawa Scale (NOS), which assesses selection of participants, comparability of study groups, and outcome assessment. Studies were categorized as low, moderate, or high quality according to their total NOS score.

Statistical Analysis

Meta-analyses were performed using Review Manager (RevMan, version 5.4). For continuous outcomes, standardized mean differences (SMDs) with 95% confidence intervals (CIs) were calculated, while dichotomous outcomes were analyzed using odds ratios (ORs) with 95% CIs. A fixed-effects model was applied unless substantial heterogeneity was present, in which case a random-effects model was used. Heterogeneity was assessed using the chi-square (χ²) test and the I² statistic, with I² > 50% indicating moderate to high heterogeneity. Publication bias was explored through funnel plot inspection and Egger’s regression test, where applicable.

Results

Search and Study Selection

**Figure 1:**
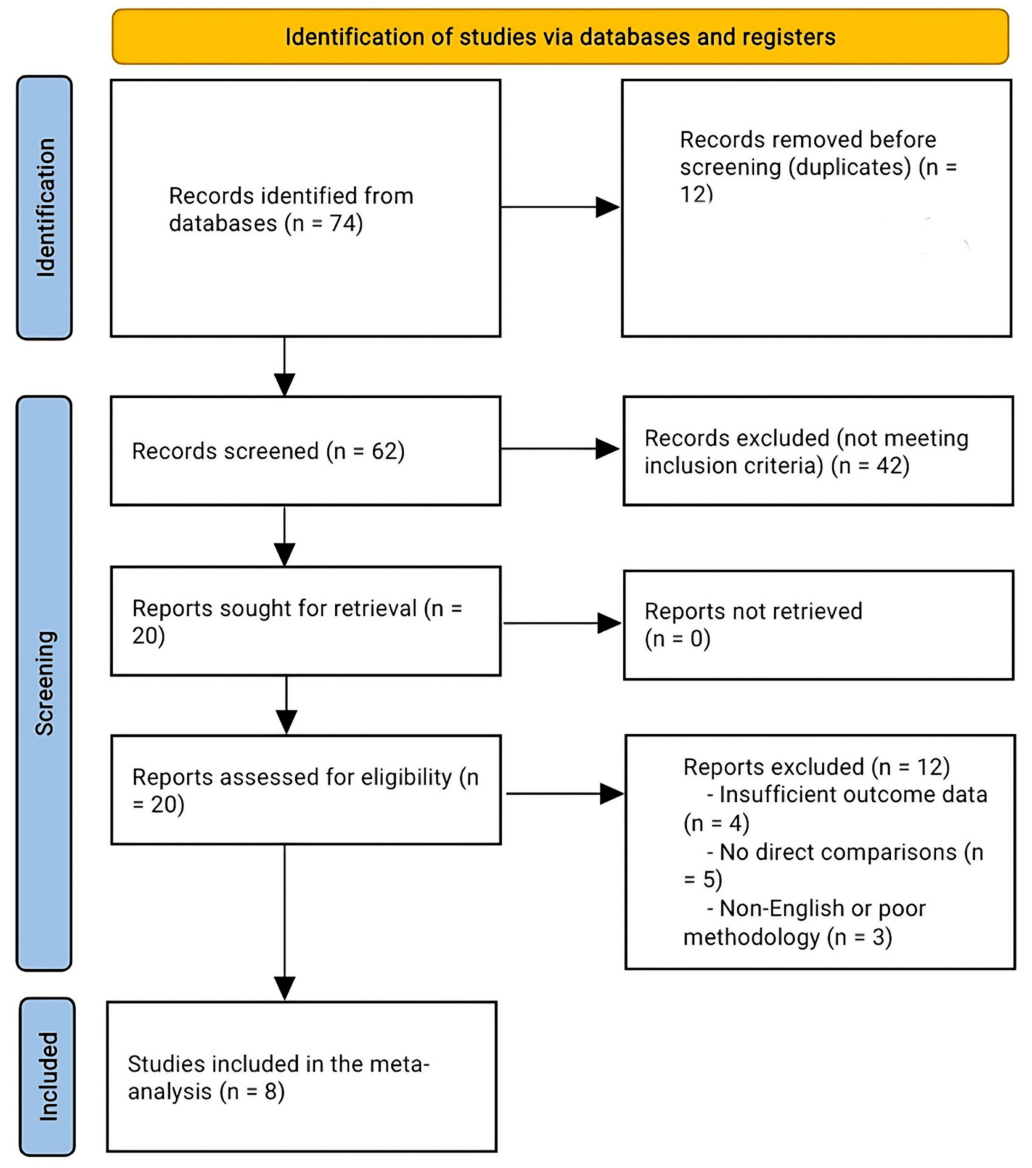
PRISMA flow chart for the included studies The PRISMA flow diagram summarizes the study selection process. The systematic search identified 74 records, with 12 duplicates removed. After screening 62 titles and abstracts, 42 studies were excluded for not meeting eligibility criteria (non-comparative design, inappropriate fracture classification, or lack of relevant outcomes such as MEPS, DASH, ROM, or complications). Twenty full-text articles were assessed, and 12 were excluded due to non-comparative design, insufficient data, language restrictions, or methodological limitations. Eight studies were included in the final quantitative synthesis (meta-analysis). DASH, Disabilities of the Arm, Shoulder and Hand; MEPS, Mayo Elbow Performance Score; ROM, range of motion

The systematic search identified 74 records. After removal of 12 duplicates, 62 articles remained for title and abstract screening. Of these, 42 were excluded because they did not meet eligibility criteria, primarily due to non-comparative study design, inappropriate fracture classification, or the absence of relevant clinical outcomes such as MEPS, DASH, ROM, or complications. A total of 20 full-text articles were assessed for eligibility, with 12 excluded for reasons including non-comparative design, insufficient data, language restrictions, or methodological limitations. Ultimately, eight studies met all inclusion criteria and were included in the quantitative synthesis (meta-analysis). The PRISMA flow diagram (Figure 1) summarizes the screening and selection process. All studies that met the predefined inclusion and exclusion criteria were included, and no eligible studies were excluded.

Study Characteristics

This meta-analysis included eight comparative cohort studies evaluating RHA versus ORIF for Mason type III and IV radial head fractures, encompassing a total of 457 patients with balanced treatment group distribution. Most studies were level III evidence, comprising both retrospective and prospective cohort designs. Participants were adults aged 20-81 years, with varying gender and activity levels. Fractures were predominantly comminuted, with several studies including complex injuries such as the terrible triad, elbow dislocations, and ligamentous damage. All studies adhered to the Mason classification, focusing on type III (severely comminuted) and type IV (fractures with dislocation). Surgical techniques varied: ORIF commonly used locking plates and screws, while RHA employed modular or monopolar prostheses. Treatment selection was influenced by fracture comminution, patient age, and surgeon preference. Follow-up durations ranged from 12 months to over seven years, allowing evaluation of both short- and mid-term outcomes. Primary outcome measures included the MEPS, DASH score, visual analog scale (VAS) for pain, and ROM assessments. Some studies also reported using QuickDASH, Patient-Rated Elbow Evaluation (PREE), and the Broberg and Morrey Elbow score. Overall, RHA was associated with superior early functional outcomes and greater ROM, particularly in highly comminuted or dislocated fractures. ORIF produced effective results in younger patients with less severe fragmentation, with comparable long-term outcomes in select cohorts. Reported complications included heterotopic ossification, prosthesis loosening, and fixation failure; RHA generally demonstrated lower revision rates. Key study characteristics, including design, sample size, demographics, surgical techniques, follow-up duration, outcomes assessed, and main conclusions, are summarized in Table 1.

**Table 1:** Summary of studies comparing RHA and ORIF in Mason type III and IV fractures. Source: [5,6,8,12-14,16,17] AO, Arbeitsgemeinschaft für Osteosynthesefragen (Association for the Study of Osteosynthesis); DASH, Disabilities of the Arm, Shoulder and Hand; HO, heterotopic ossification; LCP, locking compression plate; PIN, posterior interosseous nerve; MEPS, Mayo Elbow Performance Score; RHR, radial head resection; ROM, range of motion; SBI rHead, small bone innovations radial head prosthesis; TUNP, tardy ulnar nerve palsy; VAS, visual analog scale

Study	Study Design	Sample Size	Level of Evid	encePatient Demographics	Intervention Details	Follow-up Durati	onOutcome Measures	Results	Complications	Conclusions
Lorenz et al. [5]	Retrospective comparative cohort study with blinded outcome assessor	43 patients: 25 with 3–4 fragment fractures (19 ORIF, 6 RHA); 18 with >4 fragments (5 ORIF, 12 RHA)	Level III	Mean age 47.2 years (20–81); 22 males (51.2%), 21 females (48.8%)	ORIF using low-profile locking plates; RHA with modular bipolar prostheses	Mean 4.56 years (1.92–7.58)	ROM, MEPS, DASH, VAS	RHA superior in pronation/supination (p=0.043); outcomes comparable overall	ORIF: non-union, heterotopic ossification; RHA: radiolucent lines, capitulum erosion	RHA favored for >4 fragments; functional outcomes comparable
Chen et al. [14]	Retrospective cohort study	55 patients: 30 RHA, 25 ORIF	Level III	Mean age: RHA 58.6 ± 9.9, ORIF 57.8 ± 9.2; 63% male (RHA), 60% (ORIF)	ORIF with F3 mini locking plates; RHA with monopolar titanium prostheses	Mean 49.7 months (RHA), 46.9 months (ORIF)	ROM, MEPS, DASH, VAS	RHA had higher MEPS (p=0.035); ROM better; no significant difference in pain or DASH	RHA: 10% stiffness, valgus deformity; ORIF: 24% stiffness, implant failure, heterotopic ossification	RHA superior in function and ROM; similar complications; advised when fixation difficult
Pogliacomi et al. [6]	Retrospective cohort study	63 patients: ORIF (34), RHA (20), RHR (9)	Level III	Mean age 55.8 years (28–74); 71.4% male. Mason III: 38, IV: 25	ORIF (plate/screws), RHA (SBI rHead), RHR – chosen per fracture severity	Mean 52 months (12–108)	MEPS, radiographic OA, heterotopic ossification, prosthetic loosening	RHA superior in type IV (p=0.012); type III similar. Type IV, coronoid fractures predicted worse outcomes.	ORIF: infection, transient PIN palsy; RHA: radiolucency; RHR: none. No revisions.	RHA superior for type IV; similar results for type III. Coronoid fractures predicted instability
Liu et al. [16]	Retrospective cohort study	72 elderly patients: 37 RHA, 35 ORIF	Level III	Mean age 67.1 ± 1.25 (62–81); 31 males, 41 females	RHA (modular prosthesis, Wright Medical); ORIF (AO locking plates)	Mean 13.8 ± 1.9 months (RHA), 14.5 ± 1.3 (ORIF)	Broberg and Morrey Score, VAS, ROM	RHA 93.2 ± 1.4 vs ORIF 81.3 ± 1.3 (p=0.0079). VAS higher in RHA; ROM not significantly different.	RHA: no complications; ORIF: lower satisfaction but no major issues	RHA superior in short-term function for elderly; ROM similar; further long-term studies needed
Yan et al. [17]	Prospective comparative cohort study	39 patients: 20 RHA, 19 ORIF	Level III	Mean age RHA 36.5 ± 6.6, ORIF 35.5 ± 6.3; 72% male	RHA (metal monopolar prosthesis); ORIF (Synthes system); all terrible triad injuries	36 months	MEPS, ROM arcs, complications	RHA superior in MEPS (p=0.009), flexion-extension (p=0.01), and pro-supination arcs (p=0.04)	RHA: 4 complications (20%); ORIF: 9 (47.4%), including stiffness and fixation failure	RHA provided better function and fewer complications; preferred for terrible triad with comminution
Ryu et al. [12]	Retrospective comparative cohort study	42 patients: 20 RHA, 22 ORIF	Level III	Age RHA 53.2 ± 14.9, ORIF 48.0 ± 17.2; equal gender	RHA (EVOLVE/Floating prostheses); ORIF (Synthes LCP plate); single surgeon	Mean 18.2 months (15–31)	DASH, PREE, ROM, Complications	RHA better in flexion-extension arc (p=0.042) and supination (p=0.022); other outcomes comparable	RHA: stiffness, ulnar nerve palsy; ORIF: fixation failure, TUNP, stiffness	RHA and ORIF comparable overall; RHA better ROM; ORIF preferred in younger patients
Al-Burdeni et al. [13]	Retrospective cohort study	36 patients: 19 ORIF, 17 RHA	Level III	Mean age ORIF 34.1 ± 1.6, RHA 38.1 ± 2.6; mostly male (89%)	ORIF vs RHA (implant unspecified); choice per fracture severity	Mean 15 months	QuickDASH, ROM, surgical time, complications	No significant difference in QuickDASH (p=0.58) or ROM recovery (p=0.13). RHA shorter surgery time (p<0.0001).	ORIF: 4 complications (21%); RHA: 3 (18%); no revisions required	No significant difference between ORIF and RHA in outcomes; RHA faster surgery; individualized planning advised
Chen et al. [8]	Retrospective cohort study	102 patients: 34 ORIF, 34 RHA, 34 resections	Level III	All Mason type III fractures; demographics not detailed	ORIF with locking plates; RHA with prosthesis; resection as third arm	≥12 months	Broberg and Morrey Score, VAS, excellent/good rate, complications	RHA 94.2 ± 3.4 vs ORIF 86.5 ± 9.9 vs resection 80.6 ± 9.0. RHA > ORIF > resection (all p<0.05).	Resection: 47% arthritis, 5 HO; RHA: 6% minor issues; ORIF: 24% stiffness/arthritis	RHA best for pain relief, function, and lowest complications; ORIF next best; resection least effective

Quality Assessment of the Included Studies

The methodological quality of the eight included studies was evaluated using the NOS, which assesses non-randomized studies across three domains: selection of cohorts, comparability of study groups, and outcome assessment. Each study received a score out of 9, with higher scores indicating better methodological quality. Studies were categorized as low, moderate, or high quality based on their total NOS score [18]. Overall, the studies demonstrated moderate to high methodological quality. Most scored well in the selection and outcome assessment domains. Variability was observed in the comparability domain, primarily due to inconsistent adjustment for confounding factors such as patient age, fracture severity, and differences in surgical technique. A detailed summary of NOS scores for each study, including the distribution of stars across the three domains, is provided in Table 2.

**Table 2:** Quality assessment of included studies using the NOS Source: [5,6,8,12-14,16,17] *Indicates a low score for the respective category. **Represents a moderate score, reflecting acceptable quality. ***Denotes a high score, indicating strong quality in the respective domain. NOS, Newcastle–Ottawa Scale

Study	Selection	Comparability	Outcome	Total score (out of 9)
Lorenz et al. [5]	***	***	**	7
Chen et al. [14]	***	***	**	8
Pogliacomi et al. [6]	***	**	**	6
Liu et al. [16]	***	**	**	6
Yan et al. [17]	***	***	**	8
Ryu et al. [12]	***	***	**	7
Al-Burdeni et al. [13]	***	**	**	6
Chen et al. [8]	***	***	***	9

Results of meta-analysis

Comparison of RHA and ORIF for Elbow ROM

**Figure 2:**
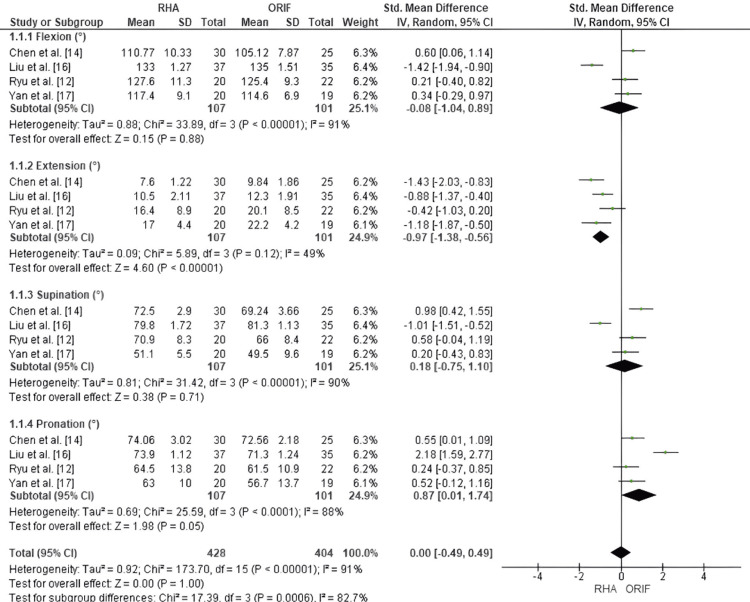
Forest plot comparing RHA and ORIF for elbow ROM outcomes in flexion, extension, supination, and pronation. Forest plot comparing postoperative elbow ROM between RHA and ORIF in Mason type III and IV fractures. RHA showed significant improvement in extension and borderline improvement in pronation, with no significant differences in flexion or supination. Pooled analysis across all ROM domains showed no overall difference between groups. Source: [12-17] ORIF, open reduction and internal fixation; ROM, range of motion; RHA, radial head arthroplasty

Postoperative elbow ROM, including flexion, extension, supination, and pronation, was compared between RHA and ORIF for Mason type III and IV fractures. In terms of flexion. no significant difference was observed between groups (SMD = -0.08; 95% CI: -1.04 to 0.89; p = 0.88), though heterogeneity was high (I² = 91%). In terms of supination, similarly, supination showed no significant difference (SMD = 0.18; 95% CI: -0.75 to 1.10; p = 0.71), with substantial heterogeneity (I² = 90%). In terms of extension, RHA demonstrated a significant advantage in postoperative extension (SMD = -0.97; 95% CI: -1.38 to -0.56; p < 0.00001), with moderate heterogeneity (I² = 49%). In terms of pronation, there was a borderline significant improvement favoring RHA (SMD = 0.87; 95% CI: 0.01 to 1.74; p = 0.05), amid high heterogeneity (I² = 88%). When pooling all four ROM domains, there was no overall significant difference between RHA and ORIF (SMD = 0.00; 95% CI: -0.49 to 0.49; p = 1.00), with high heterogeneity (I² = 91%) (Figure 2).

Publication Bias Assessment for Elbow ROM

**Figure 3:**
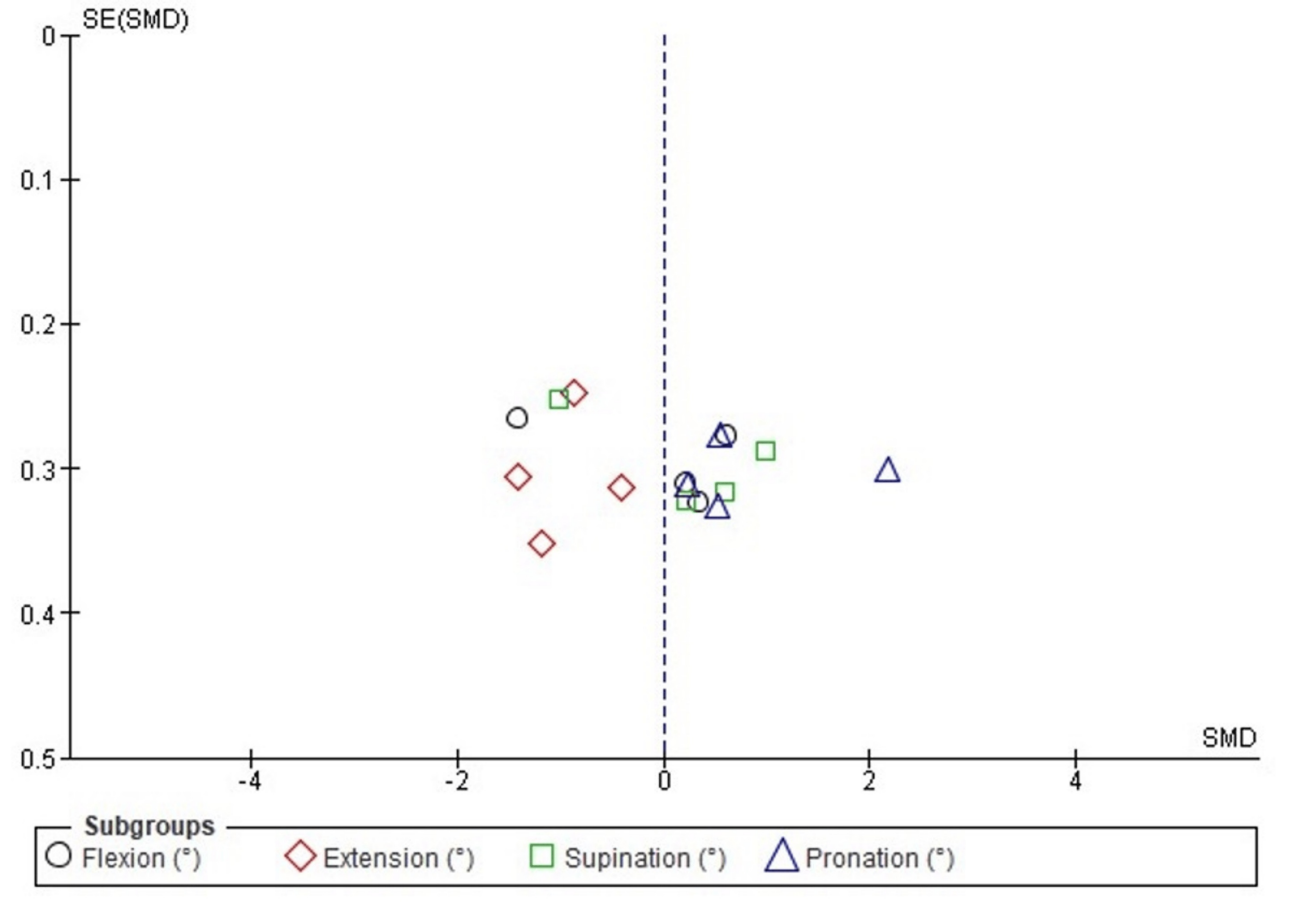
Funnel plot evaluating publication bias for ROM outcomes (flexion, extension, supination, and pronation) in studies comparing RHA and ORIF. Funnel plot assessing publication bias for postoperative elbow ROM outcomes (flexion, extension, supination, and pronation). The plot showed relative symmetry with mild dispersion, particularly in extension studies, consistent with clinical heterogeneity rather than systematic publication bias. Egger’s regression test confirmed no significant publication bias (p > 0.05). ORIF, open reduction and internal fixation; ROM, range of motion; RHA, radial head arthroplasty

Publication bias for postoperative elbow ROM outcomes (flexion, extension, supination, and pronation) was evaluated using funnel plot inspection and Egger’s regression test. The funnel plot showed a relatively symmetrical distribution of effect sizes across standard errors. Some dispersion was noted, particularly among studies reporting extension, suggesting mild asymmetry likely attributable to clinical heterogeneity or differences in surgical techniques and patient characteristics rather than systematic publication bias. Egger’s regression test confirmed the absence of significant publication bias (p > 0.05). The funnel plot for ROM outcomes is presented in Figure 3.

Elbow Function Recovery (MEPS Score)

**Figure 4:**
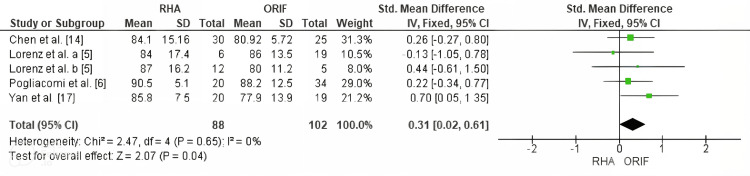
Forest plot comparing RHA and ORIF for elbow function using the MEPS. Forest plot comparing MEPS between RHA and open reduction and internal fixation (ORIF). Meta-analysis showed a significant improvement with RHA (SMD = 0.31; 95% CI: 0.02 to 0.61; p = 0.04) with low heterogeneity (I² = 0%). Source: [5,6,14,17] MEPS, Mayo Elbow Performance Score; ORIF, open reduction and internal fixation; RHA, radial head arthroplasty

Meta-analysis revealed a statistically significant improvement in elbow function with RHA compared to ORIF, as measured by the MEPS (SMD = 0.31; 95% CI: 0.02 to 0.61; p = 0.04). Heterogeneity among studies was low and not statistically significant (χ² = 2.47, degrees of freedom [df] = 4, p = 0.65; I² = 0%). The corresponding forest plot is shown in Figure 4.

Publication Bias Assessment for Elbow Function (MEPS Score)

**Figure 5:**
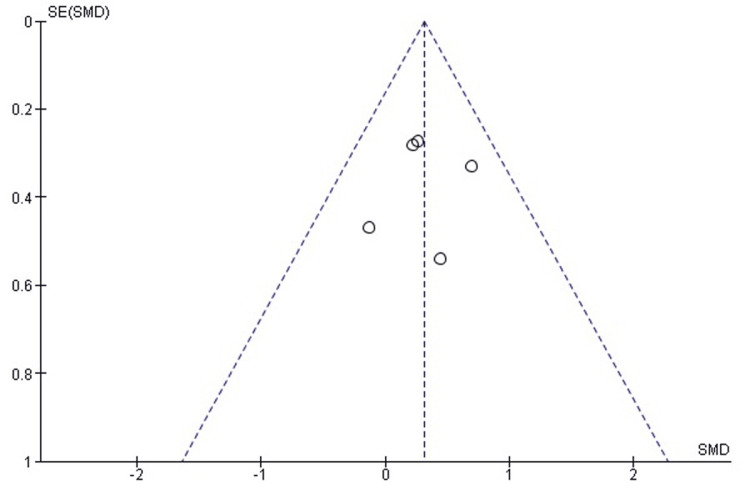
Funnel plot evaluating publication bias for elbow function (MEPS score) in studies comparing RHA and ORIF. Funnel plot assessing publication bias for MEPS outcomes. The plot showed symmetrical distribution of effect sizes with no evidence of publication bias. Egger’s regression test confirmed the absence of statistically significant bias (p > 0.05). MEPS, Mayo Elbow Performance Score; ORIF, open reduction and internal fixation; RHA, radial head arthroplasty

Publication bias for MEPS outcomes was assessed using funnel plot inspection and Egger’s regression test. The funnel plot demonstrated a symmetrical distribution of effect sizes, indicating no apparent publication bias. This finding was supported by Egger’s regression test, which did not detect statistically significant bias (p > 0.05). The funnel plot is presented in Figure 5.

Comparison of RHA and ORIF for Disability Outcome (DASH Score)

**Figure 6:**
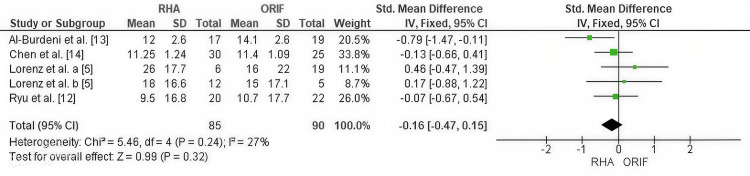
Forest plot comparing RHA and ORIF for disability outcomes using the DASH score. Forest plot comparing DASH scores between RHA and ORIF. Meta-analysis showed no significant difference between groups (SMD = -0.16; 95% CI: -0.47 to 0.15; p = 0.32) with low heterogeneity (I² = 27%). Source: [5,12,13,14] DASH, Disabilities of the Arm, Shoulder, and Hand; ORIF, open reduction and internal fixation; RHA, radial head arthroplasty

Meta-analysis of disability outcomes, measured by the DASH score, demonstrated no statistically significant difference between RHA and ORIF (SMD = -0.16; 95% CI: -0.47 to 0.15; p = 0.32). Heterogeneity among studies was low and not statistically significant (χ² = 5.46, degrees of freedom [df] = 4; p = 0.24; I² = 27%). The corresponding forest plot is shown in Figure 6.

Publication Bias Assessment for Disability Outcome (DASH Score)

**Figure 7:**
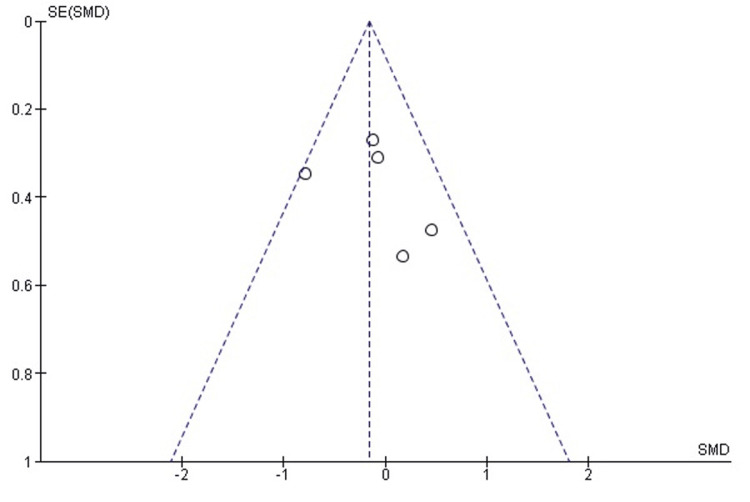
Funnel plot evaluating publication bias for DASH scores in studies comparing RHA and ORIF. Funnel plot assessing publication bias for DASH outcomes. The plot showed symmetrical distribution of effect sizes with no evidence of publication bias. Egger’s regression test confirmed the absence of statistically significant bias (p > 0.05). DASH, Disabilities of the Arm, Shoulder, and Hand; ORIF, open reduction and internal fixation; RHA, radial head arthroplasty

Publication bias for DASH outcomes was evaluated using funnel plot inspection and Egger’s regression test. The funnel plot showed a symmetrical distribution of effect sizes, indicating no significant publication bias. This was supported by Egger’s regression test, which did not detect statistically significant bias (p > 0.05). The funnel plot is presented in Figure 7.

Comparison of RHA and ORIF for Complication Rates

**Figure 8:**
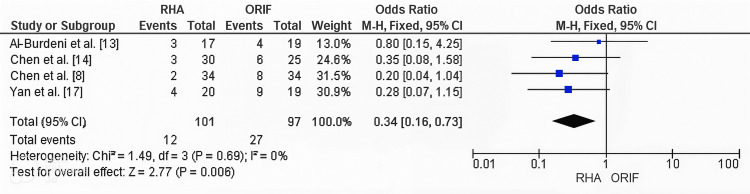
Forest plot comparing RHA and ORIF for postoperative complication rates. Forest plot comparing overall complication rates between RHA and ORIF. Meta-analysis demonstrated a significant reduction in complications with RHA (OR = 0.34; 95% CI: 0.16 to 0.73; p = 0.006) with low heterogeneity (I² = 0%). Analysis included four studies comprising 101 patients in the RHA group and 97 patients in the ORIF group. Source: [8,13,14,17] ORIF, open reduction and internal fixation; RHA, radial head arthroplasty

Meta-analysis of overall complication rates demonstrated a statistically significant reduction favoring RHA compared with ORIF (odds ratio [OR] = 0.34; 95% CI: 0.16 to 0.73; p = 0.006). Heterogeneity among the included studies was low and not statistically significant (χ² = 1.49, degrees of freedom [df] = 3; p = 0.69; I² = 0%). This analysis included four studies [8,13,14,17], with a total of 101 patients in the RHA group and 97 patients in the ORIF group. The corresponding forest plot is shown in Figure 8.

Publication Bias Assessment for Complication Rates

**Figure 9:**
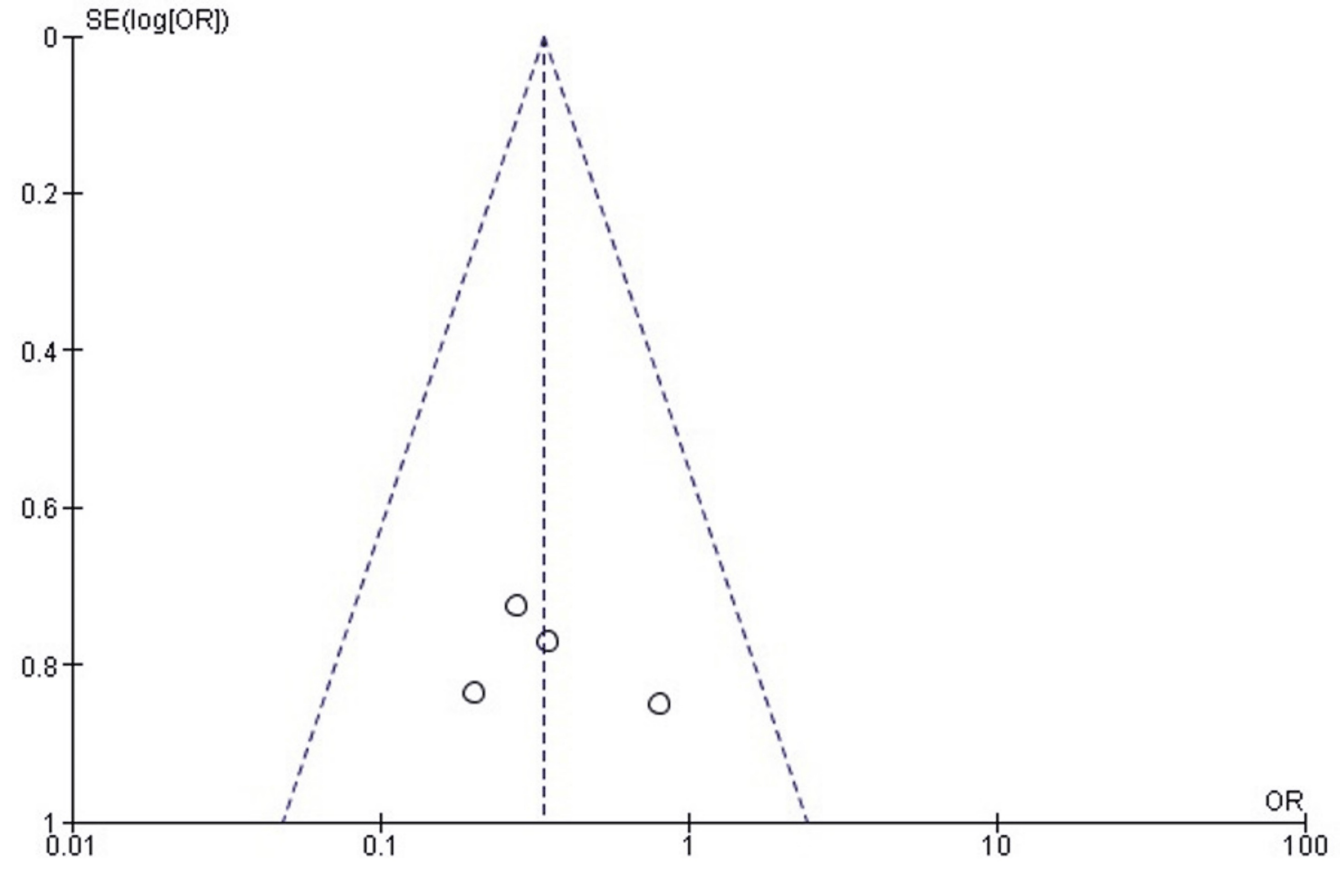
Funnel plot evaluating publication bias for complication rates in studies comparing RHA and ORIF. Funnel plot assessing publication bias for overall complication rates between RHA and ORIF. The plot showed symmetrical distribution of effect sizes, with Egger’s regression test confirming no significant publication bias (p > 0.05). ORIF, open reduction and internal fixation; RHA, radial head arthroplasty

Publication bias for overall complication rates was evaluated using funnel plot inspection and Egger’s regression test. The funnel plot appeared symmetrical, indicating no significant publication bias. This was supported by Egger’s regression test, which did not detect statistically significant bias (p > 0.05). The funnel plot is presented in Figure 9.

Discussion

The optimal surgical management of Mason type III and IV radial head fractures remains a challenging and frequently debated issue in orthopedic trauma. This systematic review and meta-analysis compared the clinical outcomes of RHA and ORIF, highlighting their respective benefits and limitations. Our findings indicate that while both procedures can achieve satisfactory functional recovery, RHA is generally associated with superior outcomes regarding complication rates, elbow mobility (particularly extension and pronation), and overall functional scores, especially in complex and comminuted fracture patterns. Emerging evidence supports the functional advantages of RHA in these severe injuries. For instance, a meta-analysis of prospective trials demonstrated significantly fewer complications and superior Broberg and Morrey Elbow scores in patients treated with RHA compared to ORIF for Mason type III fractures [11]. Similarly, Sun et al. [10] reported higher patient satisfaction, improved MEPS, and fewer mechanical failures following RHA. However, outcomes measured by the QuickDASH score were comparable between the two techniques, suggesting that patient-perceived disability may not differ substantially. The complication profiles of the two surgical strategies are notably distinct. ORIF is frequently associated with a higher incidence of mechanical failure, particularly in severely comminuted fractures [15]. In contrast, RHA tends to provide more consistent outcomes and shorter operative durations, likely due to the avoidance of complex fragment reduction and fixation.

Patient-specific factors, including age, fracture complexity, and associated soft tissue injuries, play a critical role in surgical decision-making. Ryu et al. [12] found that although RHA improved ROM across all planes, younger patients with less complex fractures often achieved better outcomes with ORIF, supporting the continued use of fixation techniques in anatomically reconstructible injuries. Fracture pattern, particularly the number of fragments, is another key determinant. Lorenz et al. [5] demonstrated that patients with four or more fracture fragments achieved significantly greater forearm rotation following RHA than ORIF, reinforcing the use of arthroplasty in markedly comminuted fractures. In Mason type IV fractures, frequently accompanied by ligamentous injuries and elbow instability, RHA appears to provide superior stabilization and long-term outcomes. Pogliacomi et al. [6] reported higher MEPS scores and better elbow stability in patients managed with RHA compared to ORIF. Nevertheless, ORIF remains a viable option in selected cases. Kaeppler et al. [19] demonstrated durable long-term outcomes with ORIF, including high union rates and satisfactory function after 14.6 years in patients with Mason type II and III fractures. The superiority of RHA in cases where ORIF or radial head excision is unfeasible is further supported by Catellani et al. [20], who observed better elbow stability and fewer complications with RHA than excision. Li and Chen [21] corroborated these findings, reporting lower complication rates (13.9%) and higher patient satisfaction (91.7%) following RHA, compared to a 58.1% complication rate in patients treated with ORIF.

Despite these advantages, concerns remain regarding prosthesis-related complications, including loosening, implant wear, and heterotopic ossification, which may necessitate revision surgery. Tarallo et al. [9] noted a 26% incidence of heterotopic ossification following RHA, although functional outcomes remained favorable in most cases. Variability in prosthesis design, surgeon experience, and postoperative rehabilitation protocols introduces further heterogeneity, potentially confounding outcome comparisons.

Importantly, most included studies were observational, of moderate methodological quality, and had relatively short follow-up durations. Consequently, the long-term durability of RHA, including prosthesis survival, late-onset instability, and progression of heterotopic ossification, remains incompletely defined. High-quality, large-scale randomized controlled trials with extended follow-up are needed to establish definitive treatment guidelines for complex radial head fractures.

Limitations

This meta-analysis is primarily limited by the predominance of retrospective cohort studies, which carry inherent risks of selection bias and confounding. Clinical heterogeneity among studies, including patient demographics, fracture severity, and associated injuries, as well as variability in surgical techniques and implant types, may affect generalizability. Inconsistencies in outcome reporting and relatively short follow-up durations limit evaluation of long-term functional outcomes and implant survival. The absence of high-quality randomized controlled trials further restricts the strength and certainty of conclusions drawn.

## Conclusions

This meta-analysis indicates that RHA provides superior functional outcomes and lower complication rates compared to ORIF for Mason type III and IV radial head fractures, particularly in complex or highly comminuted injuries. Nonetheless, ORIF remains a valid and effective option for younger patients with less severe fracture patterns in which anatomical reconstruction is feasible. Surgical decision-making should be individualized, considering patient-specific factors, fracture characteristics, and surgeon expertise. Further high-quality, prospective randomized trials with extended follow-up are warranted to strengthen the evidence base and refine treatment guidelines.
